# Chondroprotective effects of a proanthocyanidin rich Amazonian genonutrient reflects direct inhibition of matrix metalloproteinases and upregulation of IGF-1 production by human chondrocytes

**DOI:** 10.1186/1476-9255-4-16

**Published:** 2007-08-14

**Authors:** Mark JS Miller, Paul Bobrowski, Meenakshi Shukla, Kalpana Gupta, Tariq M Haqqi

**Affiliations:** 1 Center for Cardiovascular Sciences, Albany Medical College, Albany, New York, USA; 2 Rainforest Nutritionals, Inc, Raleigh, North Carolina, USA; 3 Department of Medicine, Case Western Reserve University School of Medicine, Cleveland, Ohio, USA

## Abstract

**Background:**

The Amazonian medicinal plant Sangre de grado (*Croton palanostigma*) has traditional applications for the treatment of wound healing and inflammation. We sought to characterize two extracts (progrado and zangrado) in terms of safety and oligomeric proanthocyanidin chain length. Additionally progrado was evaluated for antioxidant activity and possible chondroprotective actions.

**Methods:**

Acute oral safety and toxicity was tested in rats according under OECD protocol number 420. The profile of proanthocyanidin oligomers was determined by HPLC and progrado's antioxidant activity quantified by the ORAC, NORAC and HORAC assays. Human cartilage explants, obtained from surgical specimens, were used to assess chondroproteciton with activity related to direct inhibitory effects on human matrix metalloproteinase (MMP, gelatinolytic) activity using synovial fluid and chondrocytes activated with IL-1β (10 ng/ml). Additionally, progrado (2–10 μg/ml) was tested for its ability to maintain optimal IGF-1 transcription and translation in cartilage explants and cultured chondrocytes.

**Results:**

Both progrado and zangrado at doses up to 2000 mg/kg (po) displayed no evidence of toxicity. Oligomeric proanthocyanidin content was high for both progrado (158 mg/kg) and zangrado (124 mg/kg), with zangrado almost entirely composed of short oligomers (<6 mer), whereas the majority of oligomers in progrado exceeded 10 mers. Progrado was a remarkably potent antioxidant in the standardized tests ORAC, NORAC and HORAC. Progrado was exceptionally effective in reducing both basal and IL-1β induced glycosaminoglycan release from human cartilage explants at concentrations that also directly blocked the gelatinolytic activity of MMP-2 and MMP-9. Progrado prevented IL-1β induced suppression of IGF-1 production from human cartilage explants as well as stimulating basal IGF-1 production (P < 0.05). Comparable changes in IGF-1 gene expression were noted in cultured human chondrocytes.

**Conclusion:**

Progrado has a promising safety profile, significant chondroprotective and antioxidant actions, directly inhibits MMP activity and promotes the production of the cartilage repair factor, IGF-1. This suggests that progrado may offer therapeutic benefits in joint health, wound healing and inflammation.

## Background

There is growing interest in natural products as agents to manage health, particularly from a preventative perspective. One factor driving this interest is the advancing age of populations in developed countries and concerns over the nutrient profile and content of Westernized diets and its links to disease or poor health [1]. Nevertheless, as dietary supplements and natural products are more widely used, consumers and healthcare providers continue to require more information on their safety as well as their efficacy.

For the last decade we have been researching natural products from Amazonian and Andean cultures that are well appreciated in South America but poorly understood in other cultures. However, given the diverse and rich flora of the Amazon River basin and a strong cultural support for the use of local botanicals, they represent an exciting and largely untapped opportunity. In the present study we have focused on the traditional medicine sangre de grado, which is a dark, red latex tapped from a fast growing tree [2]. Traditionally sangre de grado is used topically for wound healing, pruritis, analgesia, and taken orally for diarrhea, ulcer healing and severe gastrointestinal distress. Hence it has broad applications within the Amazonian community. Generally without a clear mechanism of action these diverse applications are viewed with disbelief by Western societies. However, in a series of studies we have confirmed major elements of the traditional knowledge, demonstrating that oral sangre de grado accelerates the healing of gastric ulcers, prevents neurogenic-mediated intestinal mucosal secretion as a basis for its anti-diarrheal activity [3], as well as analgesic and antipruretic properties [4,5]. In terms of wound healing and inflammation sangre de grado extracts limit the transcription of a wide range of pro-inflammatory cytokines and mediators [3] and like green tea catechins promotes the apoptosis of various cancer cells [6]. There are other mechanistic studies on sangre de grado or its extracts that also support the traditional use, especially for gastrointestinal applications [7-9].

Throughout these observations we noted that a common mechanistic thread which was the ability to prevent neurogenic inflammation and specifically the activation of sensory afferent nerves [4,5]. Capsaicin acting on vanilloid receptors is the prototypical agonist for the sensory afferent nerves leading to the acute symptoms of redness, swelling, and pain [10]. The ability of sangre de grado to calm these sensory afferent nerves to a wide range of agonists (prostaglandin E_2_, protease-activated receptor 2, and capsaicin) helps explain the anti-itch, anti-nausea and analgesic properties of the medicinal plant [4,5]. Nevertheless, it is not clear if this neurogenic locus fully explains the wound healing properties of this traditional medicine and in this study we investigated other possibilities.

The chemical composition of sangre de grado has been evaluated [11-13] and one characteristic is its high proanthocyanidin content [14]. Proanthocyanidins and other chemically related polyphenols, catchins and anthocyanins, are potent antioxidants, which can modify cellular responses that are redox regulated [15]. Hence we undertook to determine the antioxidant potential of a proanthocyanidin-enriched extract of sangre de grado (Progrado^®^, Rainforest Nutritionals, Inc) using the ORAC, HORAC and NORAC assays. The oligomeric profile of progrado and the related extract, Zangrado^® ^was determined by HPLC. In addition to oral safety and toxicity, we also explored the concept that progrado may be chondroprotective.

Previously we have shown that cartilage catabolism can blocked by other polyphenols – catechins and anthocyanins [16-18]. The mechanism underlying this chondroprotective effect is their ability to suppress redox-sensitive transcription factors, thereby suppressing genes that promote catabolism and inflammation. Given that we have demonstrated that sangre de grado had anti-inflammatory and wound healing properties, and limited the expression of pro-inflammatory cytokines and enzymes, we sought to determine if progrado may affect IL-1β induced cartilage matrix breakdown and potentially, promote cartilage repair mechanisms. We have had success in identifying this pattern of responses in other South American medicinal plants that had a traditional use for treating catabolism and inflammation [19].

## Methods

### Source of medicinal plant extracts and materials

Zangrado^® ^is a proprietary lipidic extract and Progrado ^® ^is a hydrophilic based extract of sangre de grado (*Croton palanostigma*), and were obtained from Rainforest Nutritionals, Inc (Raleigh, NC), using suppliers in tropical Peru that incorporate both wild harvesting and cultivation.

Tissue culture medium and related reagents were purchased from either Mediatech (Herndon, VA) or InVitrogen (Carlsbad, CA). Recombinant human IL-1β was purchased from R&D Systems (St Paul, MN), and other chemicals were purchased from Sigma-Aldrich (Saint Louis, MO) unless otherwise noted. The extracts were dissolved in water and filtered through a 0.45 μm filter under vacuum prior to use.

### Antioxidant assays: ORAC, HORAC, NORAC

These assays were performed by Brunswick Labs (Wareham MA), the independent contract laboratory specializing in standardized antioxidant assays for food and natural products. The ORAC, HORAC and NORAC assays use the quenching of fluorescent probes as the metric for antioxidant activity. Results are standardized to a known antioxidant (trolox or gallic acid) and performed in duplicate or triplicate with results displayed as the means. Details of the assay procedure can be gleaned from Brunswick Labs website [20].

### Proanthocyanidin oligomer composition

Similar to the antioxidant assay tests, this chemical analysis was performed by the contract laboratory, Brunswick Labs and performed under Good Laboratory Practices. Total proanthocyanidin content and oligomer profile for extracts, zangrado and progrado, were determined by HPLC.

### OECD acute oral safety & toxicity

Acute oral toxicity of progrado and zangrado were evaluated separately in sprague dawley rats in compliance with OECD guidelines for testing of chemicals, Section 4, No. 420 – acute oral toxicity – fixed dose method, adopted December 17, 2001. This study was conducted in compliance with the principles of Good Laboratory Practice (GLP) as set forth in OECD Principles of Good Laboratory Practice (OECD, 1998). All tests were performed by the contract research organisation, Vedic Lifesciences, Pvt, Ltd, (Mumbai, India). A sighting study was performed prior to the main study, in which a female rat was administered the test article, suspended in water, at a dose of 300 mg/kg body weight. When no mortality or signs of toxicity were encountered in the sighting study another female rat was used for an additional sighting study at the dose of 2000 mg/kg. When no mortality or toxicity was noted the main study was commenced.

On the day of dosing all rats were frequently assessed for mortality and signs of intoxication following dosing and thereafter for 14 days. Body weights were recorded at baseline and weekly thereafter. At the end of the observational period rats were sacrificed with CO_2 _asphyxiation and subjected to a complete necropsy.

### Human cartilage explants and cultured chondrocytes

Human OA cartilage samples were procured through the Tissue Procurement Facility of University Hospitals of Cleveland/Case Western Reserve University and with prior approval of the Institutional Review Board of University Hospitals of Cleveland. The cartilage samples were obtained from patients undergoing total arthroplasty of the knee due to degenerative joint diseases. In all cases care was taken to use only "macroscopically normal" cartilage samples. No samples were exposed to radiation solely for the purpose of these studies but almost all the patients will have received X-rays as part of their clinical presentation during the execution of care. The same donor tissue was not used in all experiments but untreated controls were included in all protocols.

Chondrocytes were prepared by the enzymatic digestion of knee cartilage as previously described [16-19,21]. Chondrocytes were plated (1 × 10^6 ^cells/ml) in 35 mm culture dishes (Becton-Dickinson, Mountain View, CA, USA) and cultured in DMEM:F12 (Mediatech, Herndon, VA) supplemented with 10% FCS and 1% Penn:Strep for 72 hrs at 37°C and 5% CO_2 _in a tissue culture incubator. Chondrocytes were serum-starved overnight and then exposed progrado in a water (50 μl/ml, equivalent to 10 μg/ml)) in fresh serum-free medium for 1 hr prior to the addition of IL-1β (10 ng/ml). Cell viability before plating was monitored by the MTT assay (Cell Viability and Proliferation Assay) according to the instructions of the manufacturer (R&D Systems). In some cases, chondrocyte viability after exposure to the different agents was determined by trypan blue exclusion assay.

Full-thickness cartilage slices (20–25 mg) were dissected from the cartilage using sterile scalpel blade (Feather Safety Razor Co., Japan). Four to five cartilage pieces (approximately equal in size and weight) were transferred to each well of a 24-well, flat bottom plate (Nunc, Denmark) containing DMEM:F-12 (1:1) supplemented with antibiotics and 10% FCS and cultured for 24 hours. Subsequently the cartilage explants were cultured overnight in serum free media. The cartilage explants were treated with IL-1β alone (10 ng/ml) or with IL-1β + progrado for 72 hrs in serum free media. Explants cultured in the absence of IL-1β or progrado, were used as controls. Where appropriate, explants were exposed to progrado 15 minutes prior to the treatment with IL-1β. Total glycosaminoglycan present in the culture supernatant was estimated as described below. Two concentrations of progrado were tested in the cartilage explant studies (2 and 10 μg/ml) when compared to cultured chondrocytes (2 μg/ml). The lower dose was used in the cultured chondrocytes because of a concern that large chain length proanthocyanidin oligomers may not readily diffuse through the cartilage matrix embedding the chondrocytes in explants, which was not a constraint for cultured chondrocytes, and because the more pronounced benefits on IGF-1 production were observed at the lower dose.

### Release of GAG from cartilage explants

At the end of culture period, the culture medium was collected from each group. A 50 μl aliquot of the collected supernatant from each sample was utilized to estimate the total glycosaminoglycan (GAG) concentration by a colorimetric method employing DMMB as previously described [22]. Color intensity was read spectrophotometrically at 535 nm using the Lambda 25 spectrophotometer (Perkin-Elmer, CT) and the values were derived from a standard curve prepared using different concentrations of glucosamine sulfate. Results are expressed as micrograms of glycosaminoglycan released per milligram of cartilage tissue.

### IGF-1 production and gene expression

Total cytoplasmic RNA was prepared from primary cultures of human chondrocytes using a commercially available kit according to the instructions of the manufacturer (Qiagen, Valencia, CA). Real time quantitative RT-PCR with internal fluorescent hybridization probes was performed as previously described [17,19] and the IGF-1 gene expression was quantified using a commercially available Gene Expression Assay kit (Applied Biosystems, CA). Expression of IGF-1 mRNA was normalized to β-actin mRNA expression, and the results were expressed as fold induction relative to controls. Human IGF-1 level in cartilage explant media was quantified using a commercially available Human IGF-1 ELISA kit (R & D Systems) per manufacturer's directions.

### Inhibition of gelatinolytic enzyme activity

As gelatinases, reflecting the activity of matrix metalloproteinases, have been implicated in cartilage matrix breakdown and joint degradation in osteoarthritis, we determined the extent to which progrado directly inhibits gelatinase activity using gelatin zymography. Synovial fluid from osteoarthritis patients and culture supernatant of IL-1β-stimulated human chondrocytes was used as sources of matrix metalloproteinase activity. Human chondrocytes were prepared by the enzymatic digestion of non-affected osteoarthritis cartilage and plated at 1 × 10^6^/ml in complete medium as described above. When cultures were 80% confluent they were stimulated with IL-1β for 24 hrs in serum free medium. Culture media after this treatment was used for gelatinolytic activity. The synovial fluid and culture media were divided into 4 aliquots and resolved on gelatin containing 8.5% acrylamide gels as described [23]. The lanes were cut and incubated overnight in TCNB buffer (50 mM Tris.HCl pH 7.4, 5 mM Ca^2+^, 0.1 mM Zn^2+^, 150 mM NaCl, 0.035% Brij-35) containing either no progrado or at 1,2 and 4 μg/ml progrado. The gel was then fixed and stained with Coomassie Brilliant Blue R-350 (GE Healthcare) using standard protocols. Gelatinase activity was evident as cleared regions on a blue background. Similar results (not shown) were obtained when recombinant human MMP-9 was used. Composite images were analyzed using the Alpha-Innotech image analysis software. Band intensities (in pixels) of MMP-9 (95 kDa) or MMP-2 (72 kDa) in each lane were totaled with values in control (no Progrado) representing 100%. Percent inhibition from untreated controls was calculated using Sigma Plot software.

### Statistics and data analysis

Data was analyzed using the software package, Instat^®^, and the analysis included one way ANOVA followed by an appropriate post hoc test (Tukeys or Dunnett's). Values are expressed as mean ± SEM. Differences were considered significant at P < 0.05.

## Results

### Proanthocyanidin content and form

**Figure 1:**
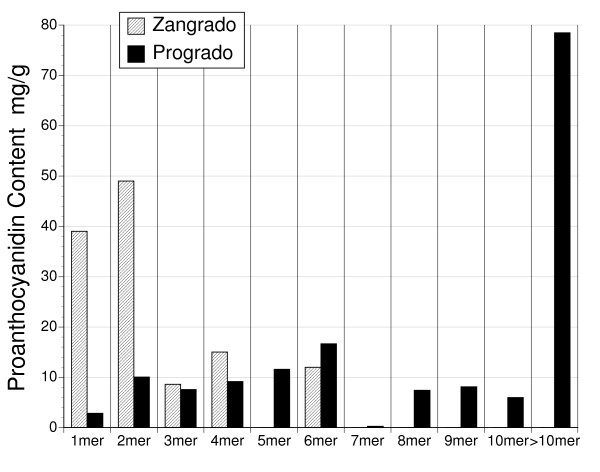
**Proanthocyanidin oligomer length for zangrado and progrado**. Oligomer length as determined by HPLC was distinctive for the two extracts, zangrado and progrado, of sangre de grado (*Croton palanostigma*). Short length oligomers were the main source of proanthocyanidins in zangrado, whereas progrado consisted of long chain oligomers. Results reflect mean values for each oligomer length for triplicate analysis.

Analysis of proanthocyanidin content and oligomer length for zangrado and progrado was performed by Brunswick Labs (Wareham, MA) by HPLC. The proanthocyanidin content was substantial in both zangrado (124 mg/g) and progrado (158.1 mg/g). Zangrado was composed of primarily short length oligomers (less than 6 mer), whereas longer length oligomers, particularly those above 10 mer in length, were present in abundance in progrado (Figure 1). This proanthocyanidin chain length difference between the two extracts may contribute to the difference in the color of these two extracts. Absorbance measured at 414 nm, where progrado was nearly 5 times greater than zangrado (data not shown), reflecting its more intense red/brown color.

### Acute oral safety and toxicity: OECD guide

Oral safety and toxicity studies for zangrado and progrado was performed by Vedic Lifesciences, Pvt. Ltd. Vedic is a contract research organization with a natural products focus. The analyses were performed in rats according to the standard protocols outlined by the OECD (Section 4, Number 420, adopted 12/2001) in accordance with the principles of Good Laboratory Practice (OECD, 1998). Both investigational agents were tested in a sighting study at 300 mg/kg, followed by another sighting study at 2000 mg/kg (for both sighting studies n = 1) and then a follow-up main study at 2000 mg/kg (n = 4).

**Figure 2:**
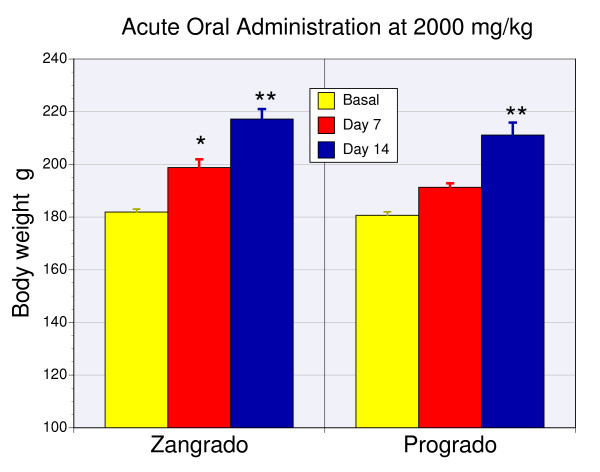
**Weight gain following a single oral dose: Acute oral toxicity**. Body weights of rats treated with either progrado (n = 5) or zangrado (n = 5) at 2000 mg/kg administered orally in accordance with OECD test 420. Weight was measured at baseline and day 7 and 14 and depicted as the mean ± sem. The * represents a significant difference from baseline (P < 0.05) and the ** indicates a significance level of P < 0.01.

Treatment with both investigational agents at these high doses did not cause weight loss, the expected result if there was systemic toxicity. Indeed, normal weight gain was observed for both progrado and zangrado (Fig. 2) following treatment at 2000 mg/kg. At the highest dose tested 2000 mg/kg, no toxicity was noted at necropsy indicated by a total lack of gross pathological alterations or clinical signs of abnormalities during the 14 day observation period. As a result both progrado and zangrado according to the Global Harmonised System (GSH) for classification of chemicals which cause acute toxicity, OECD series on testing and assessment, Number 33: has to be classified in GSH Category 5/Unclassifiable for the obligatory labeling requirement for oral toxicity. This is the highest possible safety rating in this test.

### Antioxidant assays; ORAC, NORAC and HORAC

These assays are a series of tests that evaluate the ability of agents to quench specific free radicals and oxidants. The ORAC assay is designed to measure the quenching of peroxyl radicals and uses trolox, a water-soluble vitamin E equivalent, as a reference standard. HORAC differs in that it measures the ability to quench the hydroxyl radical, with values expressed as mg gallic acid equivalents/gram. Finally the NORAC is an assay that is specific for peroxynitrite (a powerful oxidant and nitrating agent) and results are quantified in μmole of trolox equivalents/gram.

Only progrado was tested and results are summarized in Table 1. It is clear that progrado possesses a remarkable antioxidant activity across a broad range of assays. While the antioxidant activity was quantified to reference antioxidants (trolox or gallic acid) comparisons to common foods, nutraceuticals and natural products and figures can be accessed from Brunswick Labs website [20]. Briefly to aid perspective, progrado has an ORAC capacity that is 450× most nuts, 75× fresh blueberries and equivalent to powdered blueberry extracts. For the NORAC assay progrado is 21× a red wine extract and 2.3× more effective than grape seed extracts.

**Table 1:** Antioxidant profile of progrado.

Agent	ORAC μmole TE/g	HORAC mg GAE/g	NORAC μmole TE/g
Progrado	4,552	269	937

ORAC (peroxyl radical quenching) is measured in trolox equivalents/g, HORAC (hydroxyl radical quenching) in gallic acid equivalents/g, and NORAC (peroxynitrite quenching) in trolox equivalents/g. Results are expressed as means of duplicate studies.

### Chondroprotection in human cartilage explants

**Figure 3:**
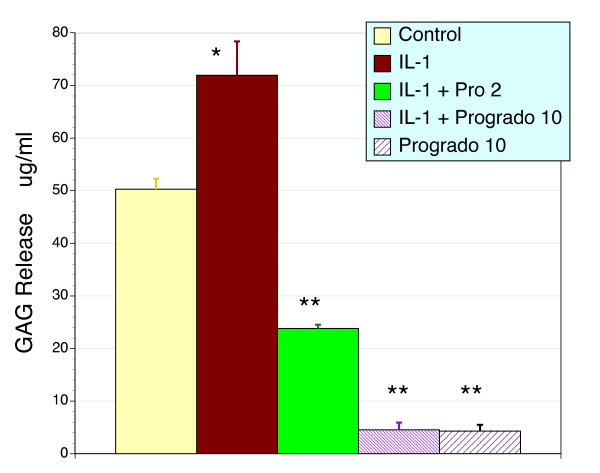
**Cartilage matrix breakdown as measured by the release of glycosaminoglycan**. Treatment of human cartilage explants with IL-1β (10 ng/ml) results in the release of glycosaminoglycans (GAG) into the media (* P < 0.01). Co-administration of progrado (2 or 10 μg/ml) blocked the IL-1β induced GAG release and resulted in a significant reduction in media levels of GAG (** P < 0.01) below the IL-1β and untreated control response. Explants treated with progrado alone (10 μg/ml) also produced a dramatic reduction in media GAG levels to that evident with progrado + IL-1β (** P < 0.01). Results are depicted as the mean ± sem.

Progrado was analyzed for its ability to modify basal and IL-1β stimulated GAG release from human cartilage explants. Progrado was able to profoundly lower basal GAG release (10 μg/ml, P < 0.01), a result that may suggest that direct inhibition of matrix metalloproteinases (Fig. 3). The addition of the pro-inflammatory, catabolic cytokine IL-1β to the explant media was able to stimulate the release of GAGs into the media (P < 0.01). This effect is thought to reflect an up-regulation of MMP production secondary to an enhanced gene expression [16-18,21]. Progrado effectively blocked IL-1β stimulated release of GAG from the cartilage explants in a dose-dependent manner (P < 0.01), and indeed reduced media GAG levels to well below baseline despite the presence of IL-1β.

### IGF-1 by Human Cartilage and Progrado

**Figure 4:**
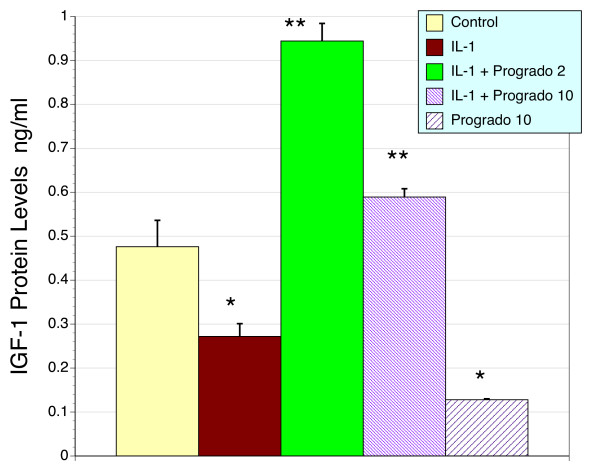
**Cartilage explant production of IGF-1 and the effects of IL-1β and progrado**. Control, untreated human cartilage explants release a defined amount of IGF-1 into the bathing media. Cartilage release of IGF-1 was significantly reduced by IL-1β (10 ng/ml, * P < 0.05). Co-treatment with progrado 2 or 10 μg/ml restored and raised IGF-1 production above basal controls (** P < 0.01). This effect was more pronounced with the lower dose of progrado (2 μg/ml) than the higher dose (10 μg/ml). In the absence of IL-1β, progrado (10 μg/ml) reduced IGF-1 production basal values (* P < 0.01). Results are depicted as the mean ± sem.

**Figure 5:**
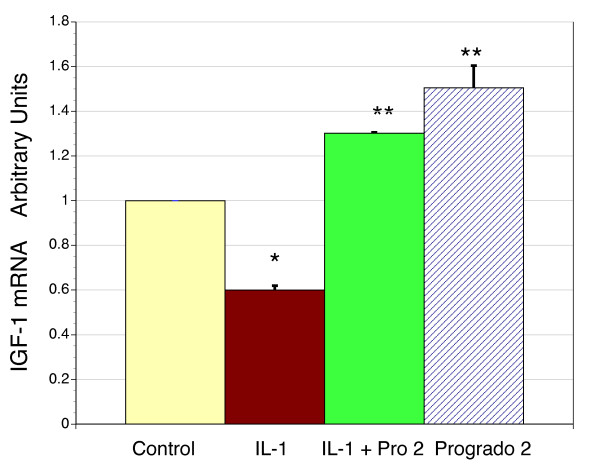
**IGF-1 mRNA levels in cultured human chondrocytes and the effects of IL-1β and progrado**. Primary cultures of human chondrocytes taken from surgical explants were examined for IGF-1 gene expression using real time RT-PCR. Basal expression was normalized to the house keeping gene β-actin. Given that lower doses of progrado were more effective in raising IGF-1 production and cultured chondrocytes do not have cartilage matrux as a barrier to agent penetration only the low dose of progrado (2 μg/ml) was evaluated. Treatment with IL-1β resulted in a significant suppression of IGF-1 mRNA levels (* P < 0.001). However, co-treatment with progrado (2 μg/ml) and IL-1β resulted in a significant increase in IGF-1 gene expression above untreated controls and IL-1β treatment (** P < 0.001). Treatment with progrado alone (2 μg/ml) was able to stimulate IGF-1 mRNA levels above all other groups (** P < 0.001). Results expressed as the mean ± SEM.

Cartilage growth and repair is largely controlled by local growth factors, the most important of which is IGF-1. During states of inflammation cartilage matrix is degraded as mimicked in Figure 4, where IL-1β stimulated GAG release in human cartilage explants (P < 0.05). During inflammation when cartilage is in a catabolic state, repair mechanisms are suppressed or rendered dormant. This is reproduced in Figure 4, where administration of IL-1β to human cartilage explants at a dose that promotes cartilage catabolism results in a suppression of IGF-1 production (P < 0.05). However, when progrado is co-administered with IL-1β, this suppressed production of IGF-1 is prevented, with normal IGF-1 production maintained at 10 μg/ml and an increase above basal values observed with the combination of IL-1β and low dose progrado (2 μg/ml, Fig. 5, P < 0.01). When high dose progrado (10 μg/ml) was incubated with unstimulated cartilage explants there is a reduction in IGF-1 production below control values (Fig. 5, P < 0.01). This indicates that while progrado may promote anabolic mechanisms responsible for repair of damaged, inflamed cartilage it appears that these actions are less pronounced at 10 than 2 μg/ml.

### IGF-1 gene expression in chondrocytes

Following the ability of progrado to promote the formation of IGF-1 production from human cartilage explants we elected to extend this observation by examining the ability of progrado to promote IGF-1 gene expression in primary cultures of human chondrocytes. Based on the observations in Figure 4, where low doses were more effective we only evaluated the low dose of progrado (2 μg/ml) in this assay. As shown in Figure 5, progrado (2 μg/ml) was able to promote the expression of IGF-1 in human chondrocytes under basal conditions (P < 0.001), confirming the data obtained with IGF-1 protein levels in cartilage explants. Consistent with the reduction of IGF-1 protein levels by IL-1β in explants, IL-1β significantly lowers IGF-1 gene expression in cultured chondrocytes (P < 0.001). Further, progrado (2 μg/ml) was effective in blocking the suppressive effects of IL-1β, and indeed raised IGF-1 mRNA levels above control levels despite the presence of IL-1β (P < 0.001). This observation also matched what was observed in terms of IGF-1 production in cartilage explants (Fig. 4). This pattern of observations indicates that restorative pathways in human cartilage that are reduced with chronic inflammation may be maintained with progrado treatment.

**Figure 6:**
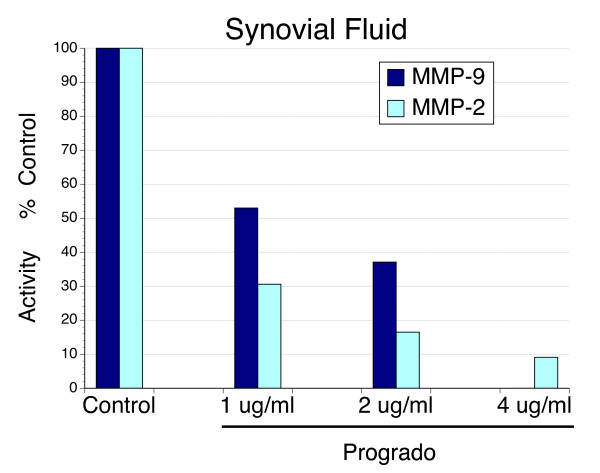
**MMP Activity from synovial fluid of subjects with osteoarthritis and the inhibitory effects of progrado**. Synovial fluid from subjects with active osteoarthritis was used as a source of matrix metalloproteinase gelatinolytic activity using gelatin zymography. Recombinant MMP-2 and MMP-9 was used for identification of gelatinolytic activity. Progrado was associated with a dose-dependent inhibition of both MMP-2 and MMP-9 activity. Complete inhibition of MMP-9 was evident at 4 μg/ml, with MMP-2 activity reduced by approximately 90%.

### Inhibition of MMP activity

**Figure 7:**
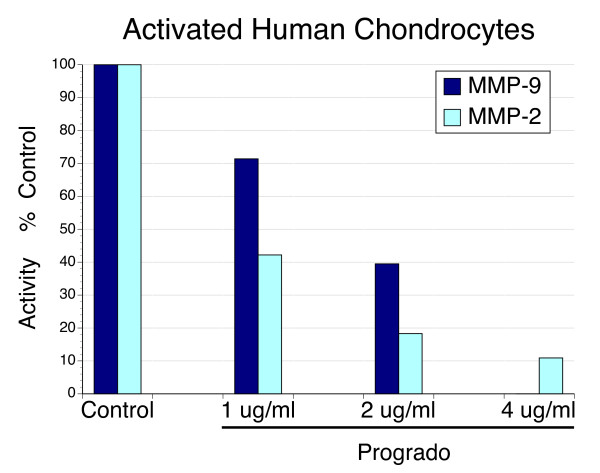
**MMP activity from culture media of IL-1β treated human chondrocytes and the inhibitory effects of progrado**. Subconfluent human chondrocytes were treated with IL-1β (10 ng/ml) to stimulate the release of matrix metalloproteinases, measured as MMP and MMP-9 using gelatin zymography. Progrado was associated with a dose-dependent inhibition of both MMP-2 and MMP-9 activity. Complete inhibition of MMP-9 was evident at 4 μg/ml, with MMP-2 activity reduced by approximately 90%.

Activity of MMP-9 was completely suppressed at progrado concentrations of 4 μg/ml, while MMP-2 activity was inhibited 90%, whether the source was synovial fluid (Figure 6) or media of activated chondrocytes (Figure 7). In contrast to the small differences in maximal inhibition by progrado on MMP-2 and MMP-9 activity, within the dose range examined in this protocol, MMP-2 activity appeared to be more sensitive to inhibition by progrado at concentrations below 4 μg/ml. These results clearly show that progrado is an effective inhibitor of the gelatinase activity present in osteoarthritis, and that this actions is demonstrated at the same concentrations of progrado that are chondroprotective and augments the chondrocyte expression and production of IGF-1.

## Discussion

In the acute oral toxicity tests both the zangrado and progrado extracts were given a GSH rating of 5 or unclassifiable. This is the top rating in this test and suggests that these extracts are particularly safe under these conditions. The parent medicinal plant sangre de grado, is traditionally used at low doses in Amazonia, as confirmed in our previous in vivo studies [3,4], and this is suggestive of a therapeutic window that is indeed large. Nevertheless, additional safety studies, especially chronic studies will be necessary to properly frame the applicability of these extracts as therapeutic agents.

The high proanthocyanidin content in both extracts is in keeping with the robust antioxidant activity. The majority of this report was focused on the profile of progrado, as zangrado has been evaluated in a number of in vitro, in vivo and clinical trial settings [3-5]. Progrado's antioxidant profile is indeed significant. The NORAC activity of progrado, which is an index of peroxynitrite quenching, was particularly exceptional. Peroxynitrite is formed from the free radicals superoxide and nitric oxide, and is thought to contribute to a wide range of pathological states [24], based on its nitrating and oxidant activities. Interestingly in healing gastric ulcers we confirmed that sangre de grado therapy reduces the expression of inducible nitric oxide synthase (iNOS) in the ulcer bed, along with IL-1β, IL-6, TNFα and COX_2_, so it may limit the tissue burden of peroxynitrite by molecular quenching (NORAC test) and by limiting the production of nitric oxide, peroxynitrite's molecular parent, by suppressing iNOS gene expression [3].

Suppressing redox related transcriptional events is a hallmark of polyphenols [14,25,26], a characteristic that we refer to as genonutrients, or nutrients that can affect gene expression. This has lead many to investigate the utility of these and related antioxidants as therapeutic agents in tissue injury and inflammation. In this study we focused on transcription related events in human cartilage to serve as a window as to the potential therapeutic applicability of progrado in osteoarthritis and related joint disorders. IL-1β is a major determinant of catabolic events in cartilage, which results from the stimulation of matrix metalloproteinases (MMPs) transcription and translation, which in turn digest the cartilage matrix. Experimentally this is then quantified in vitro by the release of GAG from cartilage explants [22].

We have previously demonstrated that the catechin, epigallocatechin gallate (EGCG) can negate cartilage IL-1β mediated cartilage breakdown by limiting MMP gene expression via a suppression of redox-regulated transcription factors like NF-κB [16,17]. This benefit was also evident in an animal model of rheumatoid arthritis [21], indicating the potential in vivo and therapeutic applicability of these events. Similarly, anthocyanin-rich pomegranate fruit extracts prevented cartilage matrix loss through transcription events [17].

Given that we have shown that catechins and anthocyanidins can protect cartilage [16-18,21] and are polyphenols that are chemically related to proanthocyanidins, we decided to evaluate the potential utility of progrado in this model of inflammation mediated joint damage. This application is slightly distinct from the traditional use which focuses on promoting skin wound healing and treating itch, pain and gastrointestinal disorders, but given the background information on composition and mechanisms of action it was deemed worthy of investigation. The present results exceeded expectations and indicate that progrado possesses within the same concentration range, a collage of activities that are potentially important for joint health and restoration.

Progrado was clearly chondroprotective as evidenced by the blocking of IL-1β induced cartilage catabolism (GAG release). Indeed GAG release was lowered below baseline values even in the presence of IL-1β. In general it is thought that IL-1β mediated matrix breakdown is due to increased MMP expression and activation in an arthritic joint, whereas basal GAG release is thought to reflect the actions of MMPs that are already present and active. Based on the ability of progrado to lower cartilage explant GAG release below baseline values, we extended the analysis to assess whether progrado directly affected MMP activity using gelatin zymography. Using the contrasting but related sources of synovial fluid from osteoarthritis subjects and the media from IL-1β activated human chondrocytes, we noted a dramatic suppression of MMP activity with progrado over the same concentration range that demonstrated chondroprotection and raised production of the anabolic repair factor, IGF-1. Individually these are important outcomes for joint health, but as a compendium of actions it is a novel and potentially important observation.

Recently we described the ability of extracts of two South American medicinal plants (Vincaria^® ^and RNI 249) from cat's claw (*Uncaria guianensis*) and maca (*Lepidium meyenii*) respectively, to exert chondroprotective actions in this human cartilage explant model [19]. These agents negated IL-1β induced GAG release as seen with EGCG and pomegranate fruit extracts but unlike progrado, they did not lower basal GAG release to the extent seen here with progrado. The combination of South American medicinal plant extracts vincaria and RNI 249, also raised the expression of the anabolic growth factor, IGF-1 in human chondrocytes, with their combined effects being additive [19]. This combination of medicinal plant extracts was recently evaluated in a positive controlled randomized clinical study in subjects with osteoarthritis, with significant benefits based on response rates and effect size (unpublished results) and has been commercialized under the name Reparagen^®^. The importance of this approach centers on the role that IGF-1 plays in repairing and regenerating cartilage [26-29]. Similarly, this present study progrado was able to maintain normal IGF-1 protein and mRNA levels despite the presence of IL-1β in human chondrocytes, indicating that progrado may potentially be disease modifying by limiting joint destruction and promoting repair.

While the present study did not measure frank repair or cartilage growth, IGF-1 is a well-known mediator of cartilage anabolism or repair [26-29]. Hui et al. [28] demonstrated that IGF-1 blocks the production of a range of MMPs in response to IL-1β which may contribute to the chondroprotective actions of progrado. However, in addition to secondary actions on catabolism via IGF-1, progrado directly inhibits MMP activity at the same concentrations that it promotes IGF-1 production by chondrocytes. Madry et al., [30] described cartilage repair by using transplanted chondrocytes that over-expressed IGF-1, which represents another exciting approach to the issue of joint repair. The suppression of repair processes like IGF-1 during inflammation may explain why glucosamine supplementation, which is a substrate used by growth factors for cartilage regeneration, has variable benefits [31,32]. It is intriguing to consider that approaches that restore these joint repair processes while limiting joint destruction may not only be a more effective alternative, but may render substrate-based approaches glucosamine and chondroitin more reliable.

## Conclusion

Extracts of *Croton palanostigma*, a traditional Amazonian medicinal plant, were found to have an excellent acute, oral safety profile and high concentrations of oligomeric proanthocyanidins. Progrado an extract enriched for long chain proanthocyanidin oligomers possessed a significant antioxidant activity and in human cartilage prevented catabolic breakdown of the cartilage matrix. The chondroprotective actions are associated with a direct inhibition of MMP activity and enhanced chondroycte expression and production of IGF-1, an anabolic growth factor known for cartilage repair. Natural products approaches like progrado, with diverse and interrelated actions on inflammation and repair mechanisms may offer an important option for managing joint health. Nevertheless further evaluation in more complex environments is needed to determine if these proanthocyanidin enriched extracts provide meaningful therapeutic value in arthritis, joint injury and repair.

## Abbreviations

IGF-1 insulin like growth factor 1

MMP matrix metalloproteinase

NF-κB nuclear factor kappa B

IL-1β interleukin 1 beta

IL-6 interleukin 6

TNFα tumor necrosis factor alpha

COX2 cyclo-oxygenase 2

GAG glycosaminoglycan

OECD organization for economic co-operation and development

ORAC oxygen radical absorbance capacity

HORAC hydroxyl radical absorbance capacity

NORAC peroxynitrite radical absorbance capacity

INOS inducible nitric oxide synthase

EGCG epigallocatchin gallate

RT-PCR reverse transcriptase polymerase chain reaction

GSH global harmonized system

HPLC high performance liquid chromatography

## Competing interests

MJSM is a member of the scientific advisory board of Rainforest Nutritionals, Inc. and has received an equity interest for these services.

PB is an owner and employee of Rainforest Nutritionals, Inc.

MS has declared that she has no competing interests.

KG has no competing interests.

TMH is a member of the scientific advisory board of Rainforest Nutritionals, Inc and has been a collaborating investigator on a grant to Rainforest Nutritionals, Inc. from the National Institutes of Health, USA.

## Authors' contributions

MJSM contributed to the study design, data analysis and manuscript preparation.

PB contributed to study design and manuscript preparation.

MS performed the experiments in human cartilage and chondrocytes.

KG performed the experiments assessing MMP activity.

TMH oversaw the performance of the cartilage and chondrocyte experiments and contributed to study design and manuscript preparation.
